# Draft Genome Sequence of *Dietzia maris* DSM 43672, a Gram-Positive Bacterium of the Mycolata Group

**DOI:** 10.1128/genomeA.00542-16

**Published:** 2016-06-09

**Authors:** Sonalli Ganguly, Guadalupe Jimenez-Galisteo, Daniel Pletzer, Mathias Winterhalter, Roland Benz, Miguel Viñas

**Affiliations:** a Department of Life Sciences and Chemistry, Jacobs University, Bremen, Germany; b Department of Pathology and Experimental Therapeutics, Laboratory of Molecular Microbiology, Medical School, University of Barcelona, Barcelona, Spain

## Abstract

Here, we report the draft genome sequence of *Dietzia maris*, known previously as *Rhodococcus maris*. It is 3,505,372 bp in size with a G+C content of 73%. The draft genome sequence will improve our understanding of *Dietzia maris* related to other mycolata species and constitutes a basic tool for exploring the cell wall proteins.

## GENOME ANNOUNCEMENT

*Dietzia maris* was initially isolated from soil, skin, and intestinal tracts of carp (*Cyprinus carpio*). The species is formed by Gram-positive cocci growing occasionally as short rods, with aerobic metabolism and are non-spore-forming (1). The appearance of its colony is circular, raised, or convex, with a glistening, deep-orange color (2). *Dietzia maris* holds a potential source of enzymes that are used in oil degradation and biosurfactant production (3), industrial fermentation, and also for the production of the antioxidant canthaxanthin (4). Recently, *Dietzia* spp. are emerging as a new group of opportunistic pathogens. *Dietzia maris* has been reported to cause bacteremia and septic shock, including prosthetic hip infection in immunocompromised patients (5–7).

Taxonomically, *Dietzia* spp. are closely related to the actinomycetes group. Actinomycetes comprise the genera *Corynebacterium*, *Mycobacterium*, *Nocardia*, *Rhodococcus*, *Tsukamurella*, and *Gordonia* (2). In these species, the cell wall contains mycolic acids of different length, depending on the species, which act as a permeability barrier for the passage of solutes (8). These mycolic acids act similarly to the outer membranes of Gram-negative bacteria. Many reports dealing with channel-forming proteins have been published for these species (9). These channel-forming proteins are responsible for the uptake of hydrophilic compounds across the mycolic acid layer.

A pore-forming protein from the cell wall of *Dietzia maris* was purified and its functionality studied in our laboratory (9). To have an insight into the structure and function of this pore-forming protein and to understand its relation to other pore-forming proteins in mycolata, we have sequenced the complete genome of *D. maris*.

For the extraction of genomic DNA, 100 ml of bacterial cells were grown in brain-heart infusion medium at 28°C in aerobic conditions for 2 to 3 days. The cells were pelleted down and were extracted with a commercially available bacterial genomic DNA isolation kit (GenElute bacterial genomic DNA kit, NA2110; Sigma-Aldrich Co., Germany). Whole DNA was sequenced by LGC Genomic (Berlin, Germany). Approximately 12 µg of genomic DNA were used for sequencing. Shotgun libraries were generated, and both ends of the DNA fragments were attached with adaptors. An Illumina MiSeq sequencer was used to sequence these libraries. Reads with final length <20 bases were discarded, and reads having more than one “N” were removed. After trimming and error correction of the sequences with Musket version 1.0.6, they were assembled and scaffolded using the Ray version 2.3.1 *de novo* assembler.

The draft genome sequence consists of 56 scaffolds with 3,505,372 bp and a G+C content of 73%. The draft genome was annotated with Prokaryotic Genome Automatic Annotation Pipeline (PGAAP) and with the Rapid Annotations using Subsystems Technology (RAST) (10) server and NCBI BLAST (11). We identified a total of 3,199 protein-coding genes, 49 tRNAs, 10 rRNAs, and 3 noncoding RNAs.

### Nucleotide sequence accession number.

The draft genome sequence of *Dietzia maris* DSM 43672 has been deposited in GenBank under the accession number LVFF00000000. The version described in this paper is the first version.
